# OP36 Horizon Scanning And Health Technology Assessment Reform In Australia: Quo Vadis?

**DOI:** 10.1017/S0266462325100949

**Published:** 2025-12-29

**Authors:** Franz Pichler, Orin Chisholm, Paul Fennessy, Nicholette Conway

## Abstract

**Introduction:**

Health systems in Australia and globally struggle to keep up with disruptive health technologies like gene therapies, gene editing, regenerative medicine, genomics, and artificial intelligence. These technologies promise better health outcomes but pose challenges due to rapid advancements, information gaps, and lack of a unified national approach. Solutions require new legal frameworks, workforce training, infrastructure investments, and government funding, supported by horizon scanning (HS) to anticipate future needs.

**Methods:**

We will provide a review of the history of HS efforts to date in Australia and outline our proposal for the establishment of a new HS function to transform Australia’s approach to new technologies from reactive to proactive. This function will integrate with post-reform health technology assessment (HTA) processes to support planning and priority setting across all levels of government, service providers, product developers, and consumers by identifying emerging technologies and outlining their benefits, risks, and operational implications over the short and long term.

**Results:**

Australia has a 17-year history of HS devices and diagnostic health technologies for HTA, investment, implementation, and disinvestment purposes, although such activity ceased during the pandemic. There is growing support for re-establishing HS, although stakeholders have diverse views on its use. A proposed HS approach aims to support planning and priority setting across federal and state/territory governments, service providers, product developers, and consumers by identifying emerging technologies and articulating the likely benefits, risks, and operational implications, including resource requirements, over near- and longer-term horizons.

**Conclusions:**

With respect to HTA, following the completion of the HTA Policy and Methods Review, the Australian government established an Implementation Advisory Group to develop a roadmap in 2025 for the government’s response to the review recommendations. We will discuss how our proposed HS function can integrate with the HS-related reforms that have been proposed under this review.

